# Effectiveness of Five-Element Regulatory Therapy for post-COVID syndrome: a retrospective cohort study

**DOI:** 10.3389/fmed.2025.1621948

**Published:** 2025-11-06

**Authors:** Ning Ding, Yunqiao Zhou, Haolin Zhang, Xiyan Xin, Yang Ye, Dong Li

**Affiliations:** 1 Department of Traditional Chinese Medicine, Peking University Third Hospital, Beijing, China; 2 Dongzhimen Hospital, Beijing University of Chinese Medicine, Beijing, China

**Keywords:** Five-Element Regulation Therapy, corona virus disease 2019, post-COVID syndrome, retrospective study, Traditional Chinese Medicine

## Abstract

**Introduction:**

Post-COVID syndrome is characterized by persistent, unexplained symptoms including chronic cough, palpitations, insomnia, and fatigue that develop following SARS-CoV-2 infection without identifiable causes. Current treatments show limited efficacy, requiring alternative options. This study aims to observe the effectiveness of Five-Element Regulation Therapy (FERT), a Traditional Chinese medicine (TCM) intervention, in managing post-COVID syndrome.

**Methods:**

A retrospective cohort study was conducted using clinical records of 127 post-COVID syndrome patients from the TCM outpatient department of Peking University Third Hospital. The participants were divided into two groups: 81 cases receiving FERT treatment were assigned to the exposure group, while 46 cases undergoing conventional TCM therapy served as the control group. The treatment duration was 2 weeks for both groups, followed by immediate follow-up. The outcomes included the clinical cure rate and clinical response rate at 2 weeks after the treatment initiation.

**Results:**

The FERT group demonstrated superior clinical outcomes, achieving a 61.7% cure rate and 88.9% response rate, significantly higher than the control group’s 21.7% (*p* < 0.001) and 67.4% (*p* < 0.01), respectively.

**Conclusion:**

This study provides preliminary evidence that FERT may be superior to conventional TCM therapy in managing post-COVID syndrome. Results should be interpreted with heightened caution due to the study’s inherent limitations.

## 1 Introduction

While the global COVID-19 pandemic has been declared over, its various sequelae continue to afflict the physical and mental health of a large number of patients in China (1–6). A systematic review covered 194 studies and 735,006 participants found that the five most common symptoms of post-COVID infection were fatigue (28.4%), pain/discomfort (29.7%), sleep disturbances (23.5%), breathing difficulties (22.6%), and activity limitations (22.3%) (7). A recent large-scale study on COVID-19 sequelae provided a systematic definition of “long COVID” for the first time (8). The research found that approximately 10% of individuals develop long COVID after infection with the Omicron variant, with up to 12 different symptoms including fatigue, cough, palpitations, hair loss, loss of sense of smell and taste, brain fog, chest pain, and changes in libido.

Current scientific understanding of long COVID suggests a complex, multi-system condition, with symptoms persisting for months or even years after the initial infection. The mechanisms remain incompletely understood but involve multiple interconnected pathways, including viral persistence (9), chronic inflammation (10), endothelial damage (11), mitochondrial (12), and autonomic nervous system dysregulation (13). Additionally, SARS-CoV-2’s broad tissue tropism via ACE2 receptors contributes to multi-organ damage, with cardiovascular, pulmonary, and metabolic sequelae being particularly prevalent (14). Emerging evidence also highlights the overlap with post-infectious syndromes, characterized by post-exertional malaise and unrefreshing sleep, with COVID-19 increasing the risk of post-infectious syndromes (15).

Current treatments for long COVID being investigated in clinical trials include non-pharmacological interventions such as physical exercise, rehabilitation therapy, and behavioral therapy, as well as pharmacological therapies like herbs, Paxlovid, and fluvoxamine (16). Psychological interventions like “awe therapy” also demonstrate benefits in reducing depression and stress (17). However, targeted and clinically proven therapies remain limited, with most current approaches focusing primarily on symptom management rather than treating the root pathological mechanisms.

Traditional Chinese Medicine (TCM) formulations including Shenhuang granule (18), JingYinGuBiao Formula (19), and Bufei Huoxue capsules (20) have demonstrated therapeutic potential in COVID-19 management within the Chinese clinical context. In the treatment of post-COVID sequelae, these TCM formulations have also exhibited significant therapeutic potential (21). Five-Element Regulation Therapy (FERT) is a set of therapeutic methods summarized by our team based on the theory of TCM constitution and clinical practice (22, 23). It classifies patients into five syndrome types (wood, fire, earth, metal, water) according to different main symptoms, and adopts different treatment approaches for groups with different syndrome types. Our research team has employed FERT for post-COVID syndrome management and observed certain therapeutic effects in case studies. However, these preliminary findings remain at the anecdotal evidence level and cannot provide evidence-based support for FERT’s clinical application in post-COVID treatment. To investigate the clinical effects of FERT in managing post-COVID symptoms, we conducted a retrospective cohort study analyzing its application at the COVID Recovery Clinic within the Department of Traditional Chinese Medicine at Peking University Third Hospital.

## 2 Materials and methods

### 2.1 Study design

The research conducted a retrospective analysis on patients with post-COVID-19 sequelae who received outpatient care at the Department of Traditional Chinese Medicine in Peking University Third Hospital. The research period extended from January 1, 2023, to May 31, 2023, during which all patients underwent TCM treatment. Patients receiving FERT intervention were assigned to the experimental group, while those undergoing conventional TCM treatment served as the control group. Patients were followed up at 2-weeks post-treatment to document symptom alleviation levels. Ethical approval was obtained from the Peking University Third Hospital Medical Science Research Ethics Committee (No. S2023541), and the study adhered to the principles of the Declaration of Helsinki.

### 2.2 Patients

Eligible patients were stratified into exposure and control groups, with FERT serving as the exposure factor-the experimental group received FERT intervention while the control group underwent conventional TCM treatment. Both patient groups maintained medication regimens for 2 weeks, with continuous treatment unless complete symptom resolution was achieved. This study imposed no gender-based restrictions on participant eligibility.

### 2.3 Inclusion and exclusion criteria

The inclusion criteria included: (1) Patients with a confirmed history of COVID-19 diagnosis verified by either PCR testing or SARS-CoV-2 antibody detection. (2) The interval between COVID-19 diagnosis and the current visit must be at least 1 month. (3) Symptoms such as cough, breathlessness, panic, palpitations, chest tightness, sleep disturbances, anxiety and depression, chronic fatigue, lumbar and knee pain can be caused by COVID infection. (4) Age ≥ 18 years.

The exclusion criteria were as follows: (1) Patients with less than 5 days of TCM treatment. (2) Patients with secondary infections. (3) Pregnant or lactating women.

### 2.4 Exposure and control

All participants in this study received TCM interventions. The experimental group was treated with FERT-based herbal formulations, while the control group received conventional TCM therapy guided by syndrome differentiation. For instance, Xiao Qing Long Tang was prescribed for cough, while Jiao Tai Wan was utilized for insomnia in the control group. The FERT group received customized Chinese herbal formulas and the control group was given either individualized herbal decoctions or standard Chinese patent medicines. Dosages followed conventional guidelines: herbal decoctions were prepared at standard therapeutic doses, while patent medicines were administered according to manufacturer-recommended dosages.

Patients in the FERT group were classified into five distinct categories according to their clinical manifestations. In the earliest stage of diseases, they are classified as Metal Disease in FERT therapy, characterized primarily by fever with accompanying symptoms including cough, headache, fatigue and decreased appetite. Respiratory symptoms, including chronic cough, viscous sputum production, and dyspnea, were classified as Earth Disease. Cardiac symptoms such as palpitations, precordial discomfort, and anxiety attacks were categorized as Fire Disease. The Wood Disease classification encompassed sleep disturbances, anxiety disorders, and depressive symptoms, while Water Disease included chronic fatigue syndrome along with lumbar and knee joint pain. The standard FERT formulations were appropriately modified based on individual symptom patterns (Table 1). Detailed compositions of the modified herbal formulations are provided in Supplementary Tables 1–4. All herbal materials were sourced from Peking University Third Hospital (Beijing, China). The treatment protocol consisted of one daily dose, administered twice daily (morning and evening) as 200 mL decoctions. The standard therapeutic course was established at 2 weeks, with a minimum required treatment duration of 5 consecutive days.

**TABLE 1:** Classification of disease patterns based on FERT therapy.

Disease type	Affected system	Primary symptoms	Corresponding formula
Metal Disease	Lung, systemic	Fever, cough, headache, fatigue, and decreased appetite	Metal Formula
Earth Disease	Lung	Chronic cough, viscous sputum production, and dyspnea	Earth Formula
Fire Disease	Cardiovascular	Palpitations, precordial discomfort, and anxiety attacks	Fire Formula
Wood Disease	Emotional	Sleep disturbances, anxiety disorders, and depressive symptoms	Wood Formula
Water Disease	Pain, systemic	Chronic fatigue, lumbar and knee joint pain	Water Formula

### 2.5 Follow-up

Clinical data were collected via outpatient follow-up consultations and retrospective medical record reviews. The dataset encompassed: (1) demographic characteristics, (2) consultation timelines, (3) comprehensive symptom profiles, (4) dates of COVID-19 diagnosis confirmation, and (5) longitudinal tracking of significant symptom improvement. Follow-up assessments were conducted at the 2-weeks post-consultation interval to document symptomatic relief outcomes.

### 2.6 Outcomes

The primary outcome measures of this study were clinical cure rate and clinical response rate. A patient was considered clinically cured if all COVID-19 sequelae completely resolved during the observation period. Clinical response was defined as symptomatic improvement with residual minor symptoms, while treatment failure was determined if symptoms showed no improvement or worsened. Patients who were lost to follow-up or non-compliant were classified as dropout cases. Clinical cure rate (%) = (Number of clinically cured cases/Total cases) × 100%. Clinical response rate (%) = [(Clinically cured cases + Cases with clinical response)/Total cases] × 100%.

### 2.7 Statistical analysis

The data was statistically analyzed using the GraphPad Prism 9.5.1 software. For normally distributed measurements, the mean ± standard deviation was reported, and independent samples *t*-test was performed. Frequencies and percentages were reported for counts, and *x*^2^ test was used. Fisher’s exact probability method was used if the theoretical value of T was less than 1 or the total number of samples n was less than 40. Differences were considered statistically significant at *p* < 0.05.

## 3 Results

### 3.1 Patient characteristics

**FIGURE 1:**
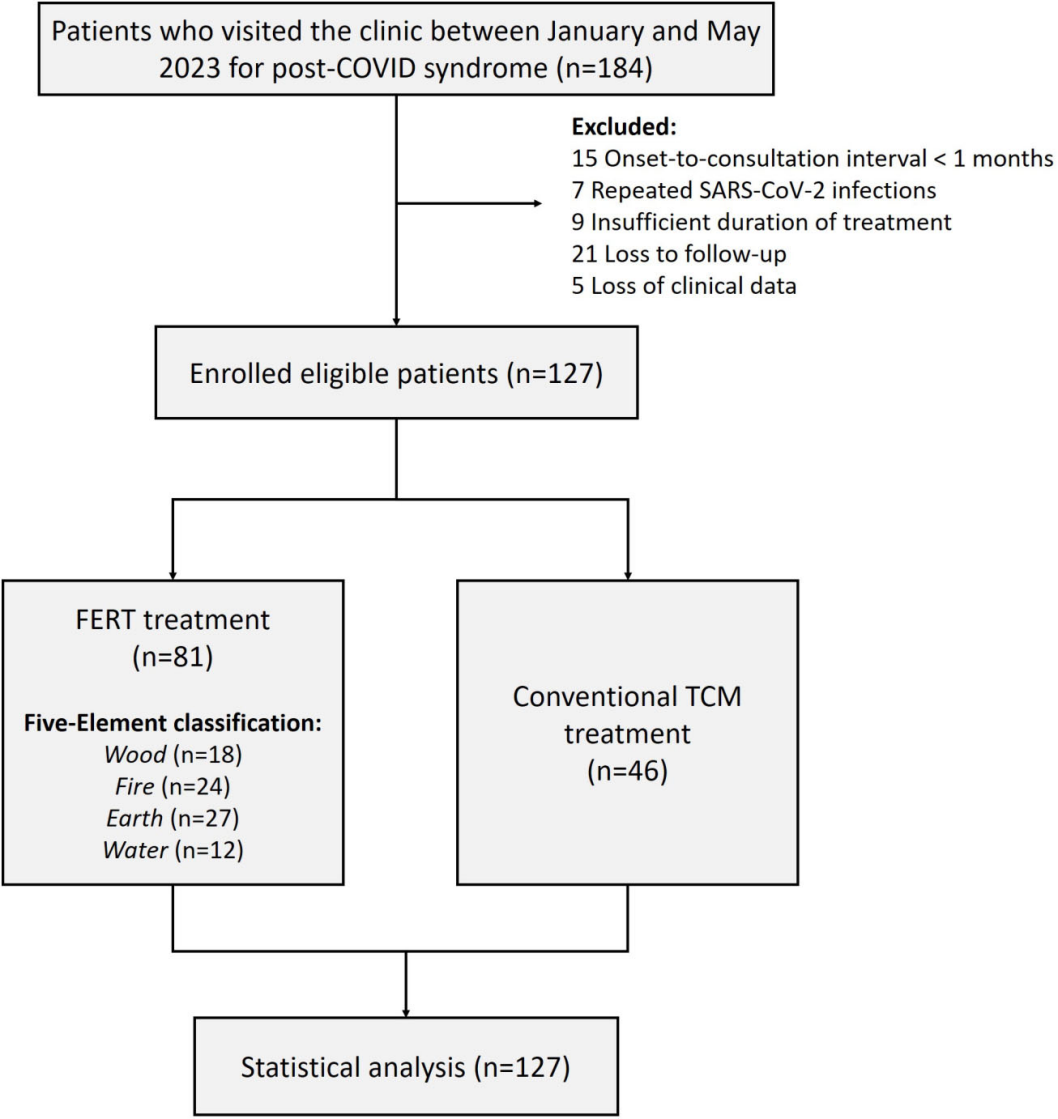
Flowchart of the study. FERT, Five-Element Regulation Therapy; TCM, Traditional Chinese Medicine.

According to the inclusion and exclusion criteria, 127 patients were eventually enrolled in the study. The study flow chart is shown in Figure 1. There were 81 cases in the FERT group and 46 cases in the control group. The baseline clinicopathological characteristics of the patients are described in Table 2. No significant differences were observed in baseline characteristics, including age, gender, and onset-to-consultation interval between the two groups (*p* = 0.984, 0.953, and 0.931, respectively).

**TABLE 2:** Comparison of clinical baseline data at enrolment between the two groups of patients.

Clinical characteristic		All participants (127)	Control group (46)	FERT group (81)	*P*-value
Age (years, $\bar{x}\pm$ s)		50.09 ± 15.54	50.13 ± 16.37	50.07 ± 15.15	0.984
Age (percent, %)	≤45	62 (48.8)	22 (47.8)	40 (49.4)	0.946
	46∼60	34 (26.8)	12 (26.1)	22 (27.2)	–
	≥61	31 (24.4)	12 (26.1)	19 (23.5)	–
Sex (percent, %)	Male	41 (32.3)	15 (32.6)	26 (32.1)	0.953
	Female	86 (67.7)	31 (67.4)	55 (67.9)	–
Onset-to-consultation interval (days)	–	62.69 ± 37.11	63.07 ± 25.41	62.47 ± 42.50	0.931
	Male	64.46 ± 34.17	63.40 ± 20.74	65.08 ± 40.33	0.882
	Female	61.84 ± 38.60	62.90 ± 27.71	61.24 ± 43.79	0.849

### 3.2 Outcomes

The clinical outcomes of both groups are shown in Table 3. The clinical cure rate and response rate were significantly better in the FERT group compared to the control group (*p* < 0.001 and *p* < 0.01, respectively). Specifically, the clinical cure rate was 61.7% (50/81) and the clinical response rate was 88.9% (72/81) in the FERT group, while the clinical cure rate was only 21.7% (10/46) and the overall effective rate was 67.4% (31/46) in the control group.

**TABLE 3:** Comparison of clinical outcomes between the two groups.

Group	Cases	Clinically cured cases	Responded cases	Ineffective cases	Dropout cases	Clinical cure rate (%)	Clinical response rate (%)
Control group	46	10	21	12	3	21.7	67.4
FERT group	81	50	22	5	4	61.7^***^	88.9^**^
Total cases	127	60	43	17	7	47.2	81.1

Compared with the control group,

^**^p < 0.01,

^***^p < 0.001.

**FIGURE 2:**
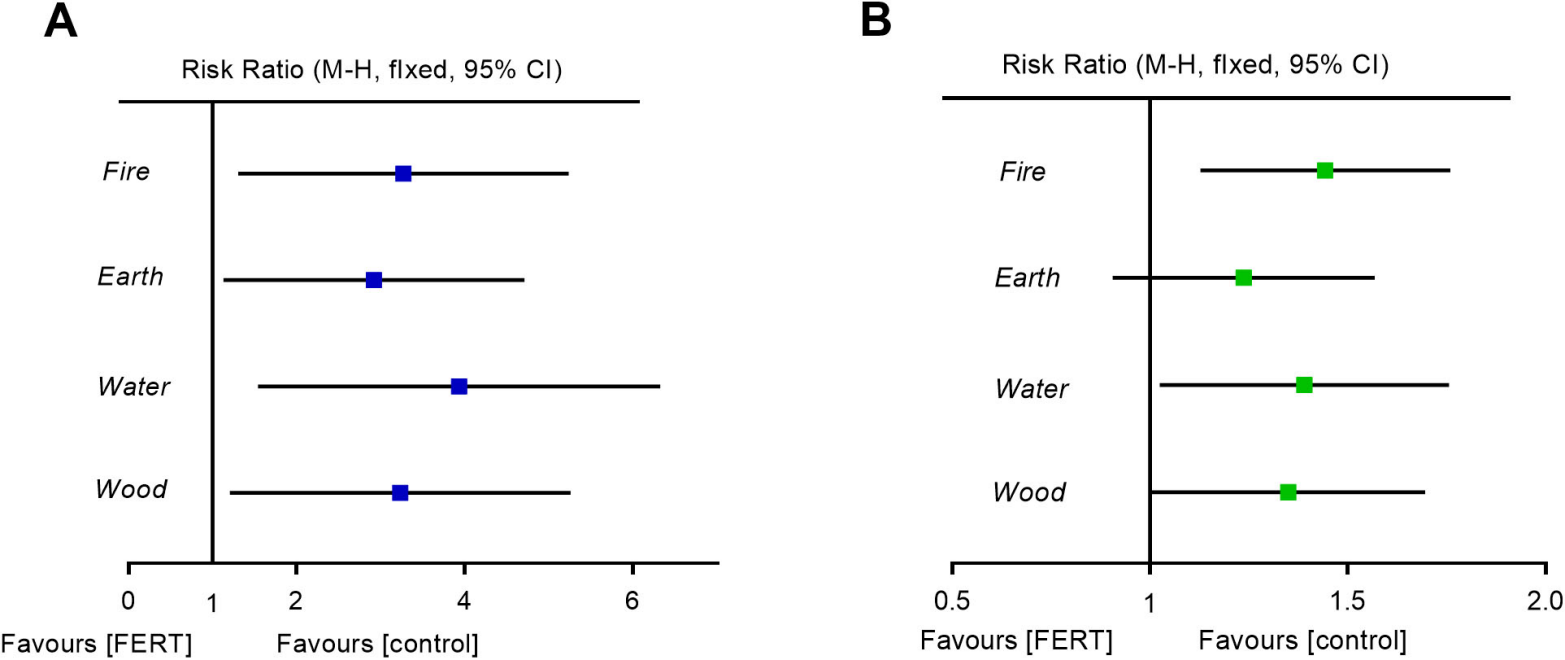
Forest plot of FERT for different syndromes. **(A)** Clinical cure rate. **(B)** Clinical response rate. CI, confidence interval; FERT, Five-Element Regulation Therapy.

In the FERT group, 33.3% (27/81) of patients had “Earth Disease,” 29.6% (24/81) had “Fire Disease,” 22.2% (18/81) had “Wood Disease,” and 14.8% (12/81) had “Water Disease “(Table 4). The order of effectiveness for different types of evidence is as follows: Water (75%), Fire (62.5%), Wood (61.1%), and Earth (55.6%). The response rates of different evidence types were compared, and the results showed that Fire (95.8%) and Water (91.7%) were the most effective, followed by Wood (88.9%) and Earth (85.2%). Compared with the control group, the clinical cure rates of all other groups have been significantly improved. In terms of clinical response rates, only the improvement in the Fire Disease group is statistically significant (*p* < 0.01; Figure 2 and Table 4).

**TABLE 4:** The efficacy of FERT in each syndrome type.

Syndrome type	Cases	Clinically cured cases	Responded cases	Ineffective cases	Dropout cases	Clinical cure rate (%)	Clinical response rate (%)
Wood	18	11	5	1	1	61.1^**^	88.9
Fire	24	15	8	1	0	62.5^***^	95.8^**^
Earth	27	15	7	2	3	55.6^**^	85.2
Water	12	9	2	1	0	75.0^***^	91.7
Control	46	10	21	12	3	21.7	67.4

Compared with the control group,

^**^p < 0.01,

^***^p < 0.001.

Table 5 shows the time intervals between patient complaints and the onset of COVID for each syndrome type. The “Earth Diseases” have the fastest onset, with an average of 38 days. This is followed by “Fire Diseases,” which have an average onset of 56 days. The “Wood Diseases” have a slightly later onset at 73 days, and the “Water Diseases” have the latest onset, with an average of 114 days.

**TABLE 5:** Comparison of the onset-to-consultation interval in each syndrome type ($\bar{x}\pm$ s).

Syndrome type	Cases	Onset-to-consultation interval (days)
Earth	27	38 ± 20.65
Fire	24	56 ± 29.25
Wood	18	73.17 ± 38.99^a^
Water	12	114.42 ± 57.4^a,^ ^b,^ ^c^

*^a^*Comparison with Earth Disease group, *p* < 0.05;

*^b^*Comparison with Fire Disease group, *p* < 0.05;

*^c^*Comparison with Wood Disease group, *p* < 0.05.

## 4 Discussion

Five-Element Regulation Therapy originates from TCM constitution theory and is a therapeutic method developed through our team’s clinical practice. During the COVID-19 outbreak in Beijing in 2022, the Department of TCM at Peking University Third Hospital established a dedicated COVID clinic. In its later stages, this clinic treated a large number of patients suffering from post-COVID sequelae. Based on the symptom profiles observed in this patient population, our team applied FERT as a treatment. However, the comparative effectiveness of FERT versus conventional TCM treatment remained unclear. This study employed a retrospective cohort design to analyze the differences in efficacy between FERT and conventional TCM therapy. The findings suggest that FERT may be more effective than conventional TCM treatment for alleviating post-COVID symptoms.

During the early battle against COVID-19 in Wuhan in 2020, TCM experts identified the disease as a “cold-dampness syndrome,” providing direction for its TCM treatment (24–27). In our clinical observations of Beijing patients, we identified distinct manifestations at different stages of post-COVID sequelae. Initially, most patients self-medicated, with some developing cough. Subsequently, patients primarily sought medical attention for cough and dyspnea. Later, their chief complaints shifted toward panic attacks, chest tightness, and palpitations. In later stages, psychological symptoms such as insomnia, irritability, anxiety, and depression were frequently observed. Some patients progressed to develop chronic fatigue, dry mouth, low-grade fever, or even reproductive dysfunction. These manifestations align with the TCM Five Elements Theory, and the data confirmed the progression sequence (Metal → Earth → Fire → Wood → Water), supporting our hypothesis. Patients were categorized into one of the Five Phase groups, and tailored body recuperative formulas were designed accordingly.

Five-Element Regulation Therapy employs five distinct prescriptions corresponding to the five symptom patterns. In this study, we observed the effects of four prescriptions, but data for the Metal Formula is lacking. This is because the Metal Formula, derived from a modified Qingfei Paidu Decoction (28, 29), primarily targets early-stage infection symptoms, including fever, cough, headache, and fatigue. However, as most patients self-administered antipyretic drugs during the initial COVID-19 phase, subsequent fever symptoms were generally mild. Consequently, the application of this specific formula in the outpatient setting was limited, resulting in an insufficient number of patients categorized under the Metal phase for observation.

The potential reasons for FERT’s superiority over conventional TCM treatment lie in its innovative integration of the dynamic pattern differentiation framework from TCM Five Elements theory with the unique pathological progression of post-COVID sequelae. The therapy precisely categorizes symptom progression into five sequential syndrome patterns according to the generative cycle of the Five Phases: Early-stage pulmonary symptoms (Metal Disease, mean 38 days), Persistent respiratory impairment (Earth Disease, mean 38 days), Cardiovascular disturbances (Fire Disease, mean 56 days), Emotional dysregulation (Wood Disease, mean 73 days), Systemic exhaustion in advanced stages (Water Disease, mean 114 days). FERT employs tailored prescriptions targeting the core pathogenesis of each stage, with individualized modifications based on specific symptoms. This temporally stratified intervention strategy effectively aligns with the characteristic sequential multi-system damage observed in post-COVID sequelae. In contrast, conventional TCM’s static pattern differentiation fails to capture this dynamic progression. Consequently, FERT demonstrates significant advantages, evidenced by a 75% clinical cure rate for the Water Disease and a 95.8% response rate for the Fire Disease – markedly higher than the control group. These benefits are particularly pronounced in middle-to-late-stage patients.

We acknowledge several limitations in this study. First, as an observational retrospective cohort study, it is inherently prone to selection bias and confounding factors, which may compromise the validity of results. Retrospective data reliance on existing records may lack standardized documentation for some variables, increasing information bias and distorting FERT-outcome associations. Non-randomized design also leaves baseline group differences unbalanced, causing residual confounding. Second, the small sample size, especially when stratified by TCM syndrome types, reduces statistical power to detect subgroup differences and raises type II error risk. Third, lacking long-term follow-up beyond 2 weeks hinders conclusions on FERT’s durability and long-term efficacy. Fourth, the single-tertiary-hospital outpatient population in Beijing limits generalizability to other regions, settings, or ethnic groups. Fifth, the primary endpoints of observation were all based on subjective symptom reports, lacking objective indicators. Finally, the absence of placebo control prevents isolating FERT’s specific effects, as other factors may contribute.

## 5 Conclusion

This retrospective cohort study investigated the potential role of FERT in managing post-COVID-19 sequelae. The results demonstrated that FERT may be superior to conventional TCM therapy in improving post-COVID symptoms including chronic cough, fatigue, insomnia, and pain. However, given the study’s retrospective design and limitations such as the lack of a blank control group, these findings should be interpreted with caution. Future high-quality studies are warranted to further analyze the therapeutic effects of FERT.

## Data Availability

The raw data supporting the conclusions of this article will be made available by the authors, without undue reservation.
