# Supplementary material for: Effectiveness of Five-Element Regulatory Therapy for post-COVID syndrome: a retrospective cohort study
Source: Front Med (Lausanne). 2025 Nov 6;12:1621948. doi: 10.3389/fmed.2025.1621948 (PMC12631303; doi:10.3389/fmed.2025.1621948)
Supplement: Supplementary file 1 [file Table_1.DOCX]

**Supplementary Materials**

**Table S1.** Detailed information of herbs in *Earth Formula*.

| **Chinese name** | **Amounts (g)** |
| --- | --- |
| *Mimahaung* | 5 |
| *Shengshigao* | 10 |
| *Zhigancao* | 6 |
| *Chaoxingren* | 6 |
| *Zexie* | 9 |
| *Baizhu* | 9 |
| *Fuling* | 15 |
| *Chaihu* | 10 |
| *Huangqin* | 6 |
| *Ziyuan* | 9 |
| *Xixin* | 3 |
| *Shengshanyao* | 12 |
| *Zhishi* | 6 |
| *Chenpi* | 12 |
| *Qingbanxia* | 9 |
| *Zhuling* | 9 |
| *Kuandonghua* | 9 |
| *Shegan* | 9 |
| *Huoxiang* | 9 |
| *Ganjiang* | 9 |
| *Donglingcao* | 15 |
| *Jinyinhua* | 15 |
| *Juhong* | 15 |
| *Cangzhu* | 10 |
| *Baiqian* | 10 |

**Table S2.** Detailed information of herbs in Fire Formula.

| **Chinese name** | **Amounts (g)** |
| --- | --- |
| *Fupian* | 5 |
| *Xixin* | 3 |
| *Mimahuang* | 3 |
| *Chenpi* | 12 |
| *Juhong* | 15 |
| *Fuling* | 15 |
| *Zhigancao* | 10 |
| *Guizhi* | 6 |
| *Xiebai* | 10 |
| *Zhiyuanzhi* | 15 |
| *Danshen* | 10 |
| *Chaosuanzaoren* | 30 |
| *Chishao* | 15 |
| *Chuanxiong* | 10 |
| *Yuanhu* | 10 |
| *Maidong* | 10 |
| *Gansong* | 10 |
| *Yinxingye* | 10 |
| *Chaihu* | 10 |
| *Hongjingtian* | 10 |
| *Donglingcao* | 15 |
| *Shouwuteng* | 10 |

**Table S3.** Detailed information of herbs in Wood Formula.

| **Chinese name** | **Amounts (g)** |
| --- | --- |
| *Fupian* | 5 |
| *Xixin* | 3 |
| *Mimahuang* | 3 |
| *Chaihu* | 10 |
| *Huangqin* | 10 |
| *Nvzhenzi* | 10 |
| *Hanliancao* | 10 |
| *Ciwujia* | 10 |
| *Shengbaizhu* | 15 |
| *Fuling* | 15 |
| *Chishao* | 15 |
| *Tusizi* | 15 |
| *Huangqi* | 15 |
| *Dangshen* | 10 |
| *Yiyiren* | 30 |
| *Baihuasheshecao* | 15 |
| *Kushen* | 15 |
| *Donglingcao* | 15 |
| *Chaosuanzaoren* | 30 |
| *Hongjingtian* | 10 |
| *Shouwuteng* | 10 |
| *Sanqifen* | 3 |

**Table S4.** Detailed information of herbs in Water Formula.

| **Chinese name** | **Amounts (g)** |
| --- | --- |
| *Chaihu* | 10 |
| *Huangqin* | 10 |
| *Nvzhenzi* | 10 |
| *Hanliancao* | 10 |
| *Ciwujia* | 10 |
| *Shengbaizhu* | 15 |
| *Fuling* | 15 |
| *Chishao* | 15 |
| *Tusizi* | 15 |
| *Huangqi* | 15 |
| *Dangshen* | 10 |
| *Yiyiren* | 30 |
| *Baihuasheshecao* | 15 |
| *Kushen* | 15 |
| *Donglingcao* | 15 |
| *Shudi* | 20 |
| *Shengshanyao* | 15 |
| *Jiuyurou* | 20 |
| *Chaosuanzaoren* | 30 |
| *Hongjingtian* | 10 |
| *zexie* | 10 |
| *Danpi* | 6 |
| *Sanqifen* | 3 |
| *Shouwuteng* | 10 |
